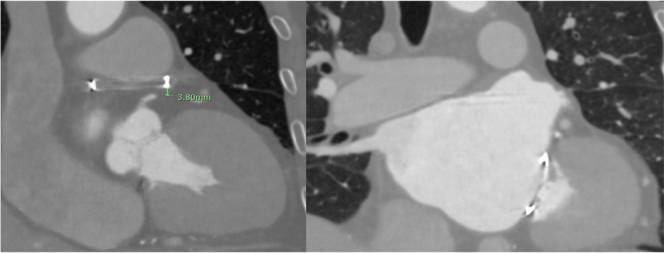# Incidence and Determinants of Incomplete Left Atrial Appendage Occlusion During Robot-Assisted Mitral Valve Surgery

**DOI:** 10.1016/j.atssr.2025.09.023

**Published:** 2025-10-23

**Authors:** Masaya Hirayama, Satoshi Kainuma, Kizuku Yamashita, Naonori Kawamoto, Kota Suzuki, Takashi Kakuta, Ayumi Ikuta, Rieko Kutsuzawa, Satsuki Fukushima

**Affiliations:** Department of Cardiovascular Surgery, National Cerebral and Cardiovascular Center, Osaka, Japan

## Abstract

**Background:**

Epicardial clipping devices are used for left atrial appendage (LAA) occlusion to prevent stroke. However, the rate and determinants of incomplete occlusion have not been determined. We clarified the rate of incomplete LAA occlusion and identified its morphologic determinants during robot-assisted minimally invasive surgery.

**Methods:**

Between 2018 and 2024, 129 patients underwent LAA occlusion with a clip device during robot-assisted mitral surgery. Of them, 45 patients (median age, 67 years; 58% male) who underwent postoperative contrast-enhanced computed tomography were enrolled. The incidence of incomplete LAA occlusion (defined as contrast material detected in the LAA with stumps of >10 mm) was determined, with focus on the difference between the LAA ostium diameter and the size of the clip device.

**Results:**

The median LAA ostium diameter at baseline was 29.3 mm (26.8-32.1 mm). The incomplete LAA occlusion was observed in 12 patients (26.7%), in 2 of whom large thrombi were demonstrated in the LAA. The rate of incomplete occlusion was significantly higher for cases in which the difference between the LAA ostium diameter and the size of the clip device was <10 mm (33.3% vs 0%; *P* < .026).

**Conclusions:**

In selected patients who underwent LAA occlusion with a clip device during robot-assisted mitral surgery, approximately one-fourth showed incomplete LAA occlusion, presumably related to LAA morphology and clip device size. Although there might be some technical challenges of LAA occlusion through the transverse sinus approach, preoperative contrast-enhanced computed tomography assessment of the LAA could be employed to facilitate proper clip device selection and to reduce the risk of incomplete occlusion.

**In Short**

▪ Approximately one-fourth of patients did not achieve complete left atrial appendage (LAA) occlusion, and cases in which the difference between the LAA ostium diameter and the size of the clip device was <10 mm had a higher rate of incomplete LAA occlusion.
▪ Preoperative contrast-enhanced computed tomography assessment of the LAA could be employed to facilitate clip device selection and to reduce the risk of incomplete occlusion.
▪ Intraoperative transesophageal echocardiography evaluation of complete LAA occlusion is critical before leaving the operating room.

Atrial fibrillation (AF) is the most common cardiac arrhythmia, and stroke in patients with AF is mainly caused by cardiogenic thromboembolism. Up to 90% of embolic strokes originate from the left atrial appendage (LAA).^1^ The latest guideline suggests that LAA occlusion should be considered during elective concomitant cardiac surgery with a class I recommendation.^2^ During cardiac surgery, the LAA can be managed through the use of clipping devices, LAA exclusion devices, external suture ligation, and suture obliteration,^3^^,^^4^ although minimally invasive approaches to surgery have become more widely used. This study aimed to clarify the technical success rate of LAA occlusion by use of an LAA clipping device during robot-assisted mitral valve (MV) surgery and to analyze the morphologic characteristics of incomplete occlusion.

## Patients and Methods

A total of 129 patients underwent robot-assisted minimally invasive MV surgery with LAA occlusion by use of the AtriClip PRO device (AtriCure) at our center between December 2018 and October 2024. In November 2024, we began offering contrast-enhanced computed tomography (CECT) for patients who were followed up at our center; these patients composed our study cohort. Patients who had undergone CECT for other reasons, such as the assessment of aortic diseases, were also included. Finally, 45 patients with available follow-up CECT images were included in this study. Patients who underwent LAA occlusion by methods other than the clipping device were excluded.

### Preoperative LAA Assessment

All patients underwent transthoracic echocardiography as a routine evaluation, and all underwent transesophageal echocardiography (TEE) for investigation of MV diseases both preoperatively and intraoperatively. CECT was also performed routinely to investigate MV diseases and to evaluate atherosclerotic plaque or calcification of the whole aorta and chest wall deformities, unless otherwise contraindicated (eg, in patients with chronic kidney disease and those with allergies to contrast agents). The diameter of the LAA ostium was measured on preoperative axial CECT scans.

### Operative Strategy

We performed a biatrial maze procedure for patients with persistent or paroxysmal AF and a left atrial maze procedure for patients with paroxysmal AF who did not need tricuspid annular plication. All procedures were performed considering the severity of heart failure and surgical risks after careful discussion with members of the cardiac team. Cardiopulmonary bypass was routinely commenced through femoral artery perfusion and femoral and right internal jugular vein drainage. If mobile plaques were noted in the abdominal or descending aorta, right axillary artery perfusion was used. With respect to operative techniques for LAA clipping, the ascending aorta and main pulmonary artery were raised by a retractor instrument inserted through the transverse sinus under cardiac arrest condition. A traction stitch (4-0 polypropylene with a small felt) was placed at the tip of the most lateral lobe of the LAA. While the traction stitch was retracted, the device was inserted through the transverse pericardial sinus, manipulated parallel to the LAA base, and deployed at the base.

### Postoperative LAA Assessment

**Supplementary Figure 1:**
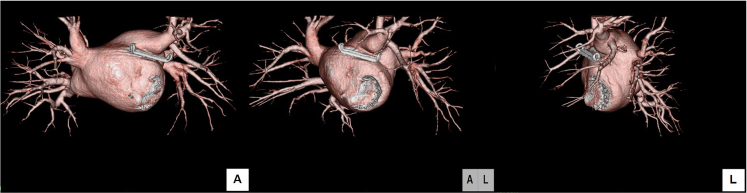


Complete occlusion was defined as nonenhancement of any remnants of the LAA or the residual stumps of the LAA being <10 mm on CECT (Supplemental Figure 1). Incomplete occlusion was defined as contrast material detected in the LAA and the stumps being >10 mm. The presence of a thrombus in the LAA was also evaluated.

### Statistical Analysis

Continuous variables are expressed as medians and interquartile ranges; categorical variables are expressed as numbers and frequencies (percentages). Between-group comparisons were performed with the Mann-Whitney *U* test for continuous variables and Fisher exact test or the *χ*^2^ test for categorical variables. Potential risk factors for incomplete LAA occlusion were identified by multivariate logistic regression analysis. All statistical analyses were conducted with R software version 4.3.1 (R Foundation for Statistical Computing). *P* values < .05 indicated statistical significance.

## Results

### Characteristics of the Patients

The median age was 67.0 years, and 26 patients (57.8%) were male. All 45 patients had AF, of whom 29 (64.4%) had persistent AF and 26 (35.6%) had paroxysmal AF. The baseline characteristics of the patients are summarized in Table 1. Operative data and early outcomes are summarized in Table 2. All patients underwent robot-assisted surgery through a right mini-thoracotomy. MV repair was performed in 43 (95.6%) patients, and MV replacement was performed in 2 (4.4%) patients. Surgical ablation was performed in all patients: biatrial maze in 38 (84.4%) and left atrial maze in 7 (15.6%) patients. Concomitant tricuspid annulus plication was performed in 27 (60.0%) patients.

**Table 1:** Baseline Characteristics of the Patients

Variable	Total (N = 45)	Complete (n = 33)	Incomplete (n = 12)	*P* Value
Age, y	67.0 (61.0-73.0)	66.0 (60.0-72.0)	68.5 (62.8-73.3)	.389
Male	26 (57.8)	18 (54.5)	8 (66.7)	.517
Persistent AF	29 (64.4)	21 (63.6)	8 (66.7)	1
Hypertension	23 (51.1)	13 (39.4)	10 (83.3)	.017
Diabetes mellitus	2 (4.4)	2 (6.1)	0	1
Coronary artery disease	2 (4.4)	1 (3.0)	1 (8.3)	.467
Chronic lung disease	6 (13.3)	3 (9.1)	3 (25.0)	.093
End-stage renal disease	1 (2.2)	1 (3.0)	0	1
LVEF, %	58.8 (52.6-63.5)	58.5 (50.1-61.0)	62.0 (55.6-66.0)	.257
LVEDD, mm	54.0 (51.0-57.0)	55.0 (51.2-57.3)	52.0 (49.0-54.5)	.172
LVESD, mm	35.0 (32.0-40.0)	37.0 (33.0-41.0)	33.0 (31.5-35.0)	.061
LAVi, mL/m^2^	79.5 (64.0-79.5)	79.0 (64.0-95.0)	82.0 (68.0-98.0)	.808

Categorical variables are presented as number (percentage). Continuous variables are presented as median (interquartile range).

AF, atrial fibrillation; LAVi, left atrial volume index; LVEDD, left ventricular end-diastolic diameter; LVEF, left ventricular ejection fraction; LVESD, left ventricular end-systolic diameter.

**Table 2:** Operative Data and Early Outcomes

Variable	Total (N = 45)	Complete (n = 33)	Incomplete (n = 12)	*P* Value
Simultaneous procedures				
MV repair	43 (95.6)	42 (97.0)	11 (91.7)	.467
MV replacement	2 (4.4)	1 (3.0)	1 (8.3)	
Tricuspid valve repair	27 (60.0)	19 (57.6)	7 (58.3)	1
Maze procedure				
Biatrial maze	38 (84.4)	29 (87.9)	9 (75.0)	.362
Left atrial maze	7 (15.6)	4 (12.1)	3 (25.0)	
LAA clipping device size				
35 mm	39 (86.7)	29 (87.9)	10 (83.3)	.650
40 mm	6 (13.3)	4 (12.1)	2 (16.7)	
Early death	0	0	0	
TIA or stroke	0	0	0	
Exploration for bleeding	0	0	0	

Values are reported as number (percentage).

LAA, left atrial appendage; MV, mitral valve; TIA, transient ischemic attack.

### Early and Late Outcomes

**Supplementary Figure 2:**
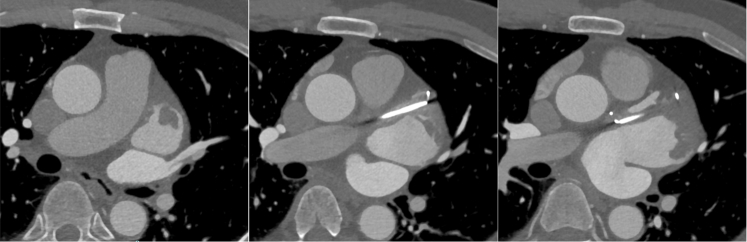


No surgical complications were observed, and no deaths occurred during follow-up. However, 2 patients (4.3%) presented with stroke at 16 and 38 months postoperatively. These 2 cerebral accidents appeared to be cardioembolic strokes as evidenced by large mobile thrombi in the LAA remnants on TEE and CECT scans (Supplemental Figure 2; Video 2). Echocardiograms at the time of readmission for stroke showed AF, and the international normalized ratios were not within the normal range despite the use of postoperative warfarin.

### Postoperative Computed Tomography Findings

**Supplementary Figure 3:**
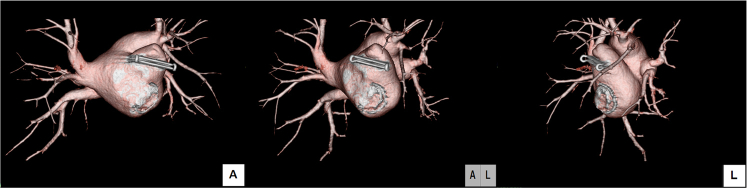


CECT scans were conducted 23.0 (9.4-38.5) months postoperatively. Complete occlusion was achieved in 33 patients (73.3%). LAA occlusion was incomplete in 12 patients (26.7%), as evidenced by residual enhanced contrast material in the LAA. Residual contrast material in the posterior part of the LAA was the typical pattern of the incomplete occlusion (Supplemental Figure 3). Of these 12 patients, 2 (4.4%) had mobile thrombi in the LAA remnants on CECT when they had stroke as mentioned earlier.

### Determinants of Incomplete LAA Occlusion

**Figure 1:**
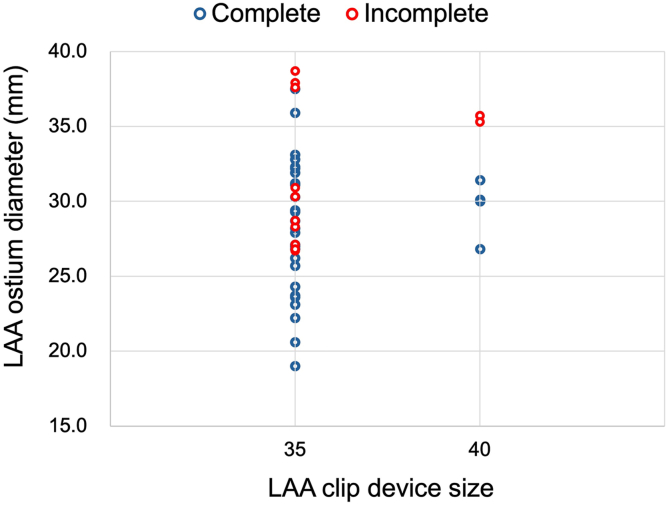
Relationship between the left atrial appendage (LAA) ostium diameter and the size of the clip device and the distribution of incomplete occlusion.

**Figure 2:**
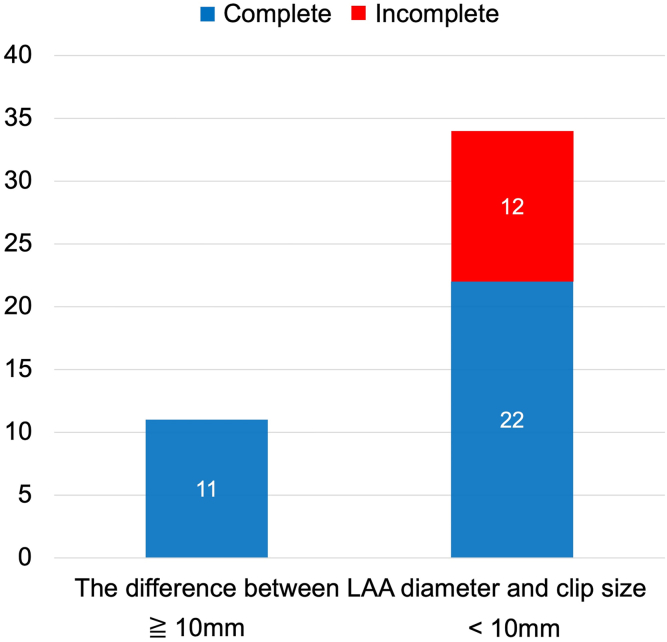
Rate of incomplete occlusion. (LAA, left atrial appendage.)

The relationship between the LAA ostium diameter and size of the clip device and the distribution of incomplete occlusion is shown in Figure 1. The rate of incomplete occlusion was 71.4% for cases in which the size of the LAA ostium diameter was >35 mm (5/7 cases). In addition, there was no case of incomplete occlusion in which the size of the LAA ostium diameter was <25 mm (0/7 cases). The difference between the LAA ostium diameter and the size of the clip device was calculated (Figure 2). Cases in which the difference was <10 mm had a higher rate of incomplete occlusion than those in which the difference was >10 mm (33.3% vs 0%; *P* < .026).

## Comment

This retrospective observational study clarified the rate of incomplete LAA occlusion with a clipping device during robot-assisted minimally invasive MV surgery. In selected patients, the rate of incomplete LAA occlusion was 26.7% on postoperative CECT, which was higher for cases in which the difference between the LAA ostium diameter and the size of the clip device was <10 mm. Of note, 2 patients with incomplete LAA occlusion presented with embolic stroke related to thrombi in the remnant LAA.

The success rate of complete LAA occlusion in this study was lower than that reported previously. Rhee and coworkers^5^ reported a complete LAA exclusion rate during minimally invasive cardiac surgery of 92.2%. This discrepancy in the complete occlusion rate may be explained by the difference in the definition of complete occlusion. Rhee and coworkers^5^ defined complete exclusion and technical success as a distance of <10 mm between the circumflex artery and clip device without leakage of contrast material or evidence of remnant pectinate muscle on the left atrial side. However, contrast material may be detected in the posterior and lower parts of the LAA in other slices on CECT even when the distance is <10 mm, suggesting that the distance of <10 mm between the circumflex artery and clipping device was not always indicative of complete occlusion (Supplemental Figure 4; Video 1). Therefore, the spatial relationship between the LAA and circumflex artery was not included in the definition.

Our data suggest that a smaller difference between the LAA ostium diameter and size of the clip device was associated with a higher rate of incomplete occlusion. We consider that some technical aspects of the procedure might be associated with the incomplete occlusion. It appeared that incomplete occlusions were primarily at the posterior inferior aspect of the LAA, which suggested that this may be due to the technical challenge of identifying the entire LAA during a transverse sinus approach, especially for the cases in which the size of the LAA ostium diameter was large. Moreover, if the LAA was fragile when the retraction stitch was applied, sufficient tension may not have been applied, or the patient’s physical frame and surgical view may also have affected the manipulation of the clip device. Our findings indicated that the device size should be at least 10 mm larger than the size of the LAA orifice. It is obvious that using a clip whose size is larger by >10 mm than the LAA orifice simply allows a larger margin of error if the clip is not placed accurately. Therefore, preoperative CECT assessment of the LAA morphology is of utmost importance and could be employed to facilitate optimal clip device selection, thereby reducing the risk of incomplete occlusion. Another crux of successful LAA occlusion is accurate intraoperative assessment of the epicardial device before leaving the operating room. Importantly, assessment of the LAA by intraoperative TEE was not performed routinely in our daily practice unless there were any technical issues with clipping procedures. However, our finding of a relatively high rate of incomplete LAA occlusion also underpins the significance of intraoperative assessment of LAA as well as MV function with TEE.

### Limitations

Two limitations of this study are the retrospective design and relatively small sample size due to the lack of routine postoperative CECT scans.

### Conclusion

In selected patients who underwent LAA occlusion with a clip device during robot-assisted MV surgery, approximately one-fourth showed incomplete occlusion of the LAA, presumably related to LAA morphology and clip device size. Although there might be some technical challenges of LAA occlusion through the transverse sinus approach, preoperative CECT assessment of the LAA could be employed to facilitate proper clip device selection and to reduce the risk of incomplete occlusion. Further follow-up with accumulated cases is necessary for analyzing the association between incomplete LAA occlusion and stroke.

## Figures and Tables

**Supplementary Figure 4:**